# Stroke mimics in patients clinically diagnosed with stroke at a tertiary teaching hospital in Tanzania: a prospective cohort study

**DOI:** 10.1186/s12883-020-01853-7

**Published:** 2020-07-07

**Authors:** Sarah Shali Matuja, Khuzeima Khanbhai, Karim M. Mahawish, Patricia Munseri

**Affiliations:** 1 Department of Internal Medicine, Catholic University of Health and Allied Sciences, P. O Box 1464, Mwanza, Tanzania; 2 Department of Cardiology, Jakaya Kikwete Cardiac Institute, Dar es Salaam, Tanzania; 3 Department of Internal Medicine, Midcentral District Health Board, Palmerston North, New Zealand; 4 Department of Internal Medicine, Muhimbili University of Health and Allied Sciences, Dar es Salaam, Tanzania

**Keywords:** Stroke, Mimics, Magnitude, Mortality, Outcomes

## Abstract

**Background:**

Stroke mimics account for up to one-third of acute stroke admissions and are a heterogeneous entity which pose diagnostic challenges. Diagnosing such patients is however crucial to avoid delays in treatment and potentially harmful medication prescription. We aimed at describing the magnitude, clinical characteristics and short-term outcomes of stroke mimics in patients clinically diagnosed with a stroke.

**Methods:**

This prospective study enrolled patients admitted with a World Health Organization clinical criteria for stroke at a tertiary hospital in Tanzania. Baseline data was collected and the simplified version of the FABS scale was used to determine its usefulness in predicting stroke mimics. The National Institute of Health Stroke Scale and Modified Rankin Scale were used to assess for admission stroke severity and outcomes respectively.

**Results:**

Among 363 patients with suspected stroke on admission, the final diagnosis was stroke mimics in 24 (6.6%) who had a mean age of 65.8 ± 15 years. Patients with stroke mimics were less likely to have cardiovascular risk factors for stroke including premorbid hypertension (7 (29.2%) vs 263 (77.6%), *p* < 0.001) and increased waist-hip ratio (9 (37.5%) vs 270 (79.6%) *p* < 0.001) for mimics and true strokes respectively. Clinical findings such as hypertension and the presence of cortical features in neurological examination occurred less in patients with stroke mimics. The simplified FABS score of ≥3 could identify patients with stroke mimics with a sensitivity and specificity of 38 and 80% respectively. The most common causes of mimics were brain tumors 6 (25%), meningoencephalitis 4 (16.7%) and epileptic seizures 3 (12.5%). The majority of patients with stroke mimics had severe disease on admission and the 30-day mortality in these patients was 54.5%.

**Conclusions:**

In the present study, the proportion of stroke mimics among patients clinically diagnosed with stroke was 6.6% and brain tumors was a common etiology. Stroke mimics were less likely to have cardiovascular risk factors and cortical signs during evaluation. We recommend further studies that can help develop clinical scales used for predicting stroke mimics in an African population.

## Background

Stroke is a clinical diagnosis as endorsed by World Health Organization (WHO), defined as rapidly developing clinical signs of focal or global disturbance in cerebral function lasting more than 24 h or leading to death with no apparent cause other than that of vascular origin [1]. It is a medical emergency and an early diagnosis is of paramount importance. This entails judicious clinical assessment supported with neuroimaging studies to confirm the diagnosis. Certain conditions however can imitate true strokes, leading to a misdiagnosis, delays and/or inappropriate treatment. Such disorders are termed stroke mimics (SM); pathological conditions that resemble stroke like clinical presentations, however are caused by diseases other than cerebrovascular disorders [2]. In modern era where thrombolytics are widely used in treating acute stroke, evidence indicates that as many as a 15% of patients treated with tissue plasminogen activator (t-PA) are SM for which the therapy is not indicated [3]. This results in unnecessary diagnostic tests, invasive procedures and longer hospital stay leading to an increased cost to the patient.

Previous data from high income states describes the proportion of SM as high as 30% of all strokes [4]. Similarly, in sub Saharan Africa (SSA), a hospital based retrospective study conducted in Nigeria, found that among 142 patients with a WHO clinical diagnosis of stroke, 23.2% had SM which included brain tumors, brain abscess, subdural hematomas, hydrocephalus and intracranial cysts [5]. Other described SM include seizures, syncope, metabolic encephalopathies (such as hypoglycemia, hyponatremia, uremia and hepatic encephalopathy), infectious disorders including meningoencephalitis and degenerative diseases amongst others [6, 7]. Notable is that, in SSA, stroke is readily diagnosed purely on clinical grounds with no confirmation due to limited access to brain imaging [8]. Therefore, in order to offer best practice, it is imperative to ensure that a timely and proper diagnosis is made to confirm stroke and exclude SM to offer optimal therapy and aim to prevent irreversible brain damage.

FABS is a proposed scale used in discriminating SM from true strokes and consists of 6 variables (1 point for each) based on clinical assessment: absence of facial droop, negative history of atrial fibrillation, age < 50 years, systolic blood pressure < 150 mmHg on admission, history of seizures and isolated sensory deficits [9]. A score of ≥3 could help identify SM with a sensitivity of 90% and specificity of 91%. More recently, the simplified FABS (sFABS) demonstrated excellent discrimination and good calibration to predict SM in a Chinese stroke population receiving t-PA [10]. In Tanzania, there is paucity of data on the magnitude and outcomes of SM. We therefore aimed at investigating the proportion of SM among patients clinically diagnosed with stroke, assess the usefulness of the sFABS scale for predicting SM and determining the short-term outcomes of SM in a tertiary teaching hospital in Dar es Salaam, Tanzania.

## Methods

### Study design and population

This prospective study was conducted at Muhimbili University of Health and Allied Science Academic Medical Center (MAMC), a tertiary public teaching hospital in Dar es Salaam, Tanzania. MAMC offers super specialized medical care to all specialties, in the capital city of Dar es Salaam and receives referred patients from both public and private hospitals from all over the country.

All study patients were enrolled from June 2018 to January 2019. Those aged ≥18 years with a WHO clinical criteria for stroke, defined as the rapidly developing clinical signs of focal or global disturbance in cerebral function lasting more than 24 h or leading to death with no apparent cause other than that of vascular origin [1] were consecutively recruited and enrolled after obtaining written informed consent. Case ascertainment was accurate as all patients presenting to the emergency department with suspected stroke are transferred directly to the medical ward. An interviewer based structured questionnaire was administered to the patients or their caregivers in the event the patient was unable to communicate. The questionnaire captured socio-demographic information, mobile numbers and past medical/drug history for hypertension, diabetes mellitus, cardiac disease. We also inquired for smoking and alcohol consumption. The date of onset of stroke symptoms and date of arrival to the hospital was recorded in the questionnaire. Physical assessment of the patients was performed and included a focused neurological examination, measurement of waist-hip circumference and blood pressure (BP) on admission. Waist-hip ratio was computed and interpreted according to the WHO guidelines: in males the ratio of ≥0.90 and females ≥0.85 was regarded as substantially increased [11]. Subsequent BP readings were spaced 5 min apart and an average blood pressure was computed. The sFABS scale consisting of 4 items with 1 point in each item present: absence of facial droop, negative history of atrial fibrillation, age < 50 years and systolic blood pressure < 150 mmHg at presentation, was used to predict SM. Similar to a previous study [10], a score of ≥3 was tested for accuracy in the diagnosis of SM. Venous blood was collected aseptically from the cubital fossae and analysis was done for blood glucose levels, chemistry profile and complete blood count. Lumbar puncture was performed in patients with suspected meningoencephalitis. Brain imaging was used to confirm the diagnosis of stroke and these were termed as true strokes with either a non-contrast brain computed tomography (CT) scan or Magnetic Resonance Image (MRI) interpreted by a senior Radiologist. Contrast was administered in those with neuroimaging findings suspicious of tumor. A 12-lead electrocardiogram (ECG) using Bionet machine was performed on the patients to look for evidence of atrial fibrillation. Stroke severity was assessed using the National Institute of Health Stroke Scale (NIHSS) on the day of admission and the Modified Rankin Scale (mRS) was used to assess for disability at 24 h, 72 h, 7 days, 14 days and 30-days post-stroke [1]. Patients were contacted by phone if they had been discharged by these time points. The study was conducted and reported using strengthening the reporting or observational studies in epidemiology (STROBE) methodology.

### Data analysis

Data was transferred from the questionnaires and entered into SPSS version 20.0 for analysis. Continuous variables were summarized and presented as means and standard deviation (SD) or medians with Interquartile Range (IQR). Comparisons of clinical presentation and 30-day outcomes among SM and true strokes were summarized as proportions and computed using Pearson’s Chi square test or Fisher’s exact test. The sFABS scale for prediction of stroke mimics was also analyzed for the purpose of determining its usefulness in the Tanzanian population. A *p* value of < 0.05 was used to denote statistical significance.

## Results

**Fig. 1:**
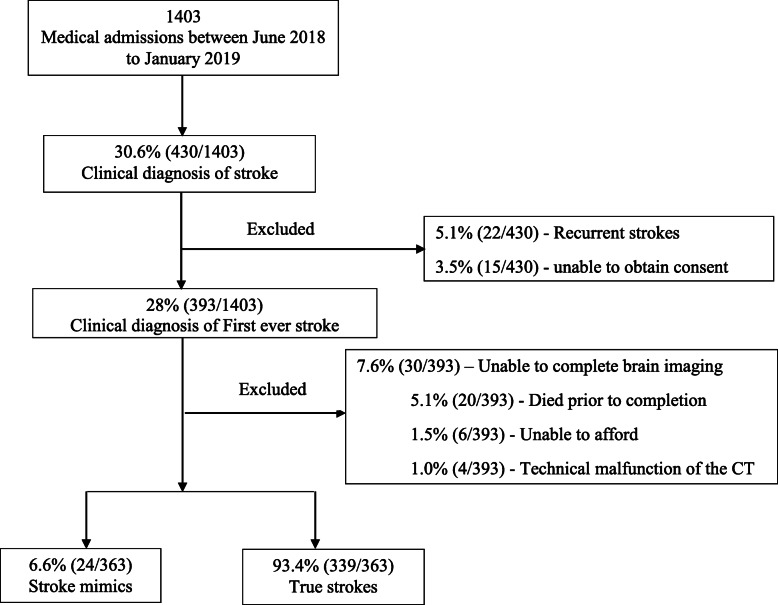
Consort diagram showing the flow of patients

**Fig. 2:**
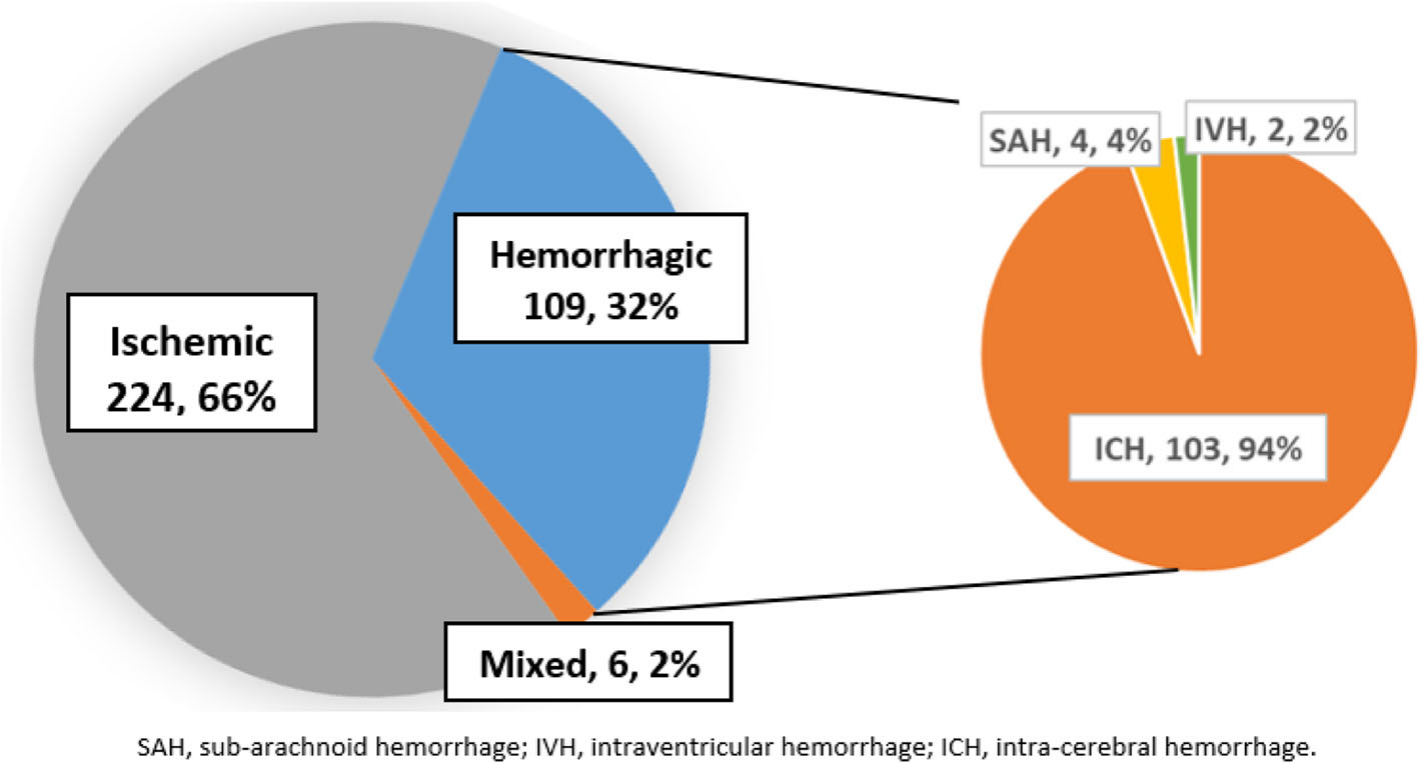
Stroke subtype among patients with true strokes

Between June 2018 to January 2019, there were a total of 1403 admissions in the general medical ward, 729 (52%) females, out of which 430 (30.6%) patients met the WHO clinical criteria for stroke. We excluded 67 (16%) of patients for the following reasons: unable to consent, experienced recurrent strokes and those who did not complete brain imaging. We recruited the remaining 363 (92.4%) with a clinical diagnosis of first ever stroke and completed neuroimaging. The proportion of SM and true strokes are summarized in Fig. 1. The distribution of stroke subtype among patients with true strokes is summarized in Fig. 2: ischemic stroke accounted for 224 (66%), 109 (32%) had hemorrhagic stroke and 6 (2%) had mixed lesions (both ischemia and hemorrhage).

### Demographic characteristics

Demographic characteristics of the SM and true strokes are summarized in Table 1. The mean age for SM and true strokes was 65.8 ± 15 and 57.9 ± 16 years, *p* = 0.024 respectively. The mean systolic and diastolic blood pressures were 126 ± 15 and 155.6 ± 21 mmHg, *p* < 0.001 and 77.2 ± 14 and 95.6 ± 11 mmHg, *p* < 0.001 for SM and true strokes respectively. The mean NIHSS score for SM and true strokes was 21.7 ± 8 and 21.2 ± 9, *p* = 0.770. SM were less likely to have premorbid hypertension 7 (29.2%) vs 263 (77.6%), *p* < 0.001 and increased waist-hip ratio 9 (37.5%) vs 270 (79.6%) *p* < 0.001 in SM and true strokes respectively.

**Table 1:** A comparison of demographic characteristics between stroke mimics and true strokes

	Stroke Mimics *N* = 24 (%)	True strokes *N* = 339 (%)	Total *N* = 363 (%)	*P* value
Age Mean ± SD	65.8 ± 15	57.9 ± 16	58.2 ± 16	0.024
Female	15 (62.5)	191 (56.3)	206 (56.7)	0.556
Ever married	24 (100)	309 (91.2)	333 (91.7)	0.242
Resident of Dar es Salaam	12 (50)	265 (78.2)	277 (76.3)	0.002
Insurance	5 (20.8)	92 (27.1)	97 (26.7)	0.5
Medical History:				
Previous Hypertension	7 (29.2)	263 (77.6)	270 (74.4)	< 0.001
Previous Diabetes	1 (4.2)	61 (18)	62 (17.1)	0.095
Previous Cardiac disease	1 (4.2)	15 (4.4)	16 (4.4)	0.714
Ever smoked	1 (4.2)	22 (6.5)	23 (6.3)	0.540
Ever consumed alcohol	5 (20.8)	68 (20.1)	73 (20.1)	0.927
Increased waist-hip ratio	9 (37.5)	270 (79.6)	279 (76.9)	< 0.001
Atrial Fibrillation	2 (8.3)	20 (5.9)	22 (7.4)	0.695
Systolic blood pressure Mean ± SD	126 ± 15	155.6 ± 21	153.7 ± 22	< 0.001
Diastolic blood pressure Mean ± SD	77.2 ± 14	95.6 ± 10	94.3 ± 12	< 0.001
NIHSS Mean ± SD	21.7 ± 8	21.2 ± 9	21.2 ± 8	0.770
Arrival to hospital from symptom onset:				
Within 1 day	12 (50)	134 (39.5)	158 (40.2)	
Day 2 to 6	11 (45.8)	168 (49.6)	194 (49.4)	0.438
> 7 days	1 (4.2)	37 (10.9)	38 (10.5)	

*SD* Standard deviation, *NIHSS* National Institute of Health Stroke Scale

### A comparison of neurological signs among patients with stroke mimics and true strokes

During neurological examination, SM were less likely to have cortical signs: extinction 4 (16.7%) vs 128 (37.8%), *p* = 0.038, hemianopia 5 (20.8%) vs 153 (45.1%), *p* = 0.020 and supra-nuclear facial paralysis 6 (25%) vs 210 (61.9%), *p* < 0.001 for SM and true strokes respectively as shown in Table 2.

**Table 2:** A comparison of neurological signs among stroke mimics and true strokes

Neurological signs	Stroke mimics *N* = 24 (%)	True strokes *N* = 339 (%)	Total *N* = 363 (%)	*P* value
Dysphasia	20 (83.3)	291 (85.8)	311 (85.7)	0.735
Dysarthria	18 (75)	285 (84.1)	303 (83.5)	0.248
Extinction	4 (16.7)	128 (37.8)	132 (36.4)	0.038
Hemianopia	5 (20.8)	153 (45.1)	158 (43.5)	0.020
Supra-nuclear facial palsy	6 (25)	210 (61.9)	216 (59.5)	< 0.001
Hemiplegia	13 (54.2)	133 (39.2)	146 (40.2)	0.184
Hemi-sensory loss	5 (20.8)	122 (36)	127 (35)	0.132
Ataxia	0 (0)	4 (1.2)	4 (1.1)	0.760

### The use of sFABS scale among patients with stroke mimics and true strokes

Table 3 summarizes the distribution of sFABS score among patients with SM and true strokes. sFABS score of ≥3 could identify patients with SM with a sensitivity and specificity of 38 and 80% respectively, while a sFABS score of ≥2 had a sensitivity of 96% and specificity of 43%.

**Table 3:** Distribution of sFABS score among patients with stroke mimics and true strokes

sFABS score	Stroke mimics *N* = 24 (%)	True strokes *N* = 339 (%)
0	0 (0)	26 (7.7)
1	1 (4.2)	120 (35.4)
2	14 (58.3)	126 (37.2)
3	8 (33.3)	54 (15.9)
4	1 (4.2)	13 (3.8)

### Causes of stroke mimics

**Fig. 3:**
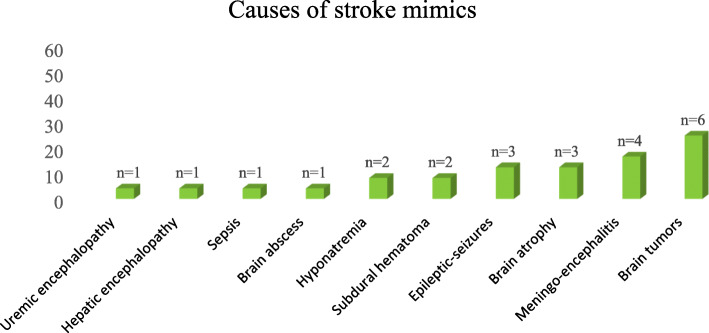
Causes of stroke mimics

The most frequent causes of SM were brain tumors 6 (25%), followed by meningoencephalitis 4 (16.7%), epileptic seizures 3 (12.5%) and brain atrophy 3 (12.5%) as summarized in Fig. 3.

### Outcomes

The overall mortality at 30 days among the 363 patients was 209 (59.8%). SM had a shorter length of hospital stay of 6.2 ± 3.5 compared to 9.1 ± 16.1 days, *p* = 0.009 in true strokes. In survivors, there were no significant differences in functional independency or mortality at 30 days (summarized in Table 4). There were 15 (4.1%) lost to follow up following discharge; 2 (8.2%) SM and 13 (3.8%) true strokes. Patients lost to follow-up were excluded from the outcome analysis.

**Table 4:** A comparison of outcomes among patients with stroke mimics and true strokes

	Stroke mimics n (%)	True strokes n (%)	Total	*P* value
Length of hospital stay	6.2 ± 3.5	9.1 ± 16.1	8.9 ± 15.9	0.009
Died at 30 days	12 (54.5)	196 (60.1)	208 (59.8)	0.295
Independency at 1 month (^a^mRS 0–2)	1 (10)	19 (14.6)	20 (14.3)	0.522

^a^*mRS* Modified Rankin Scale

## Discussion

The present study indicates that 6.6% of all admitted patients who initially met the WHO stroke definition for first event had SM as a final diagnosis. Our findings mirror those described by Forster A et al., where 6.4% of patients with suspected stroke had SM [12]. Other authors have reported higher proportions of SM (up to 30%); these variabilities in proportions could be attributed to the number of study patients enrolled, the setting and duration of the study, ours having fewer patients and a shorter study duration [3]. Nonetheless, our findings are alarming considering the majority of the countries in SSA (including Tanzania) have limited access to brain imaging. A one-year hospital based retrospective study conducted in three public tertiary hospitals in Zimbabwe included 450 patients with a clinical diagnosis of stroke. Of these, only 39.4% of the study patients underwent CT brain [13]. They suggested that lack of resources and health insurance coverage could explain this low uptake of CT scans. Therefore, this signals the utmost importance of integrating such services to facilitate an early and accurate diagnosis, particularly when stroke presentations are associated with such high mortality (approximately 60% at 30 days in this study). Wardlaw et al., found that early stroke imaging with CT is the least costly strategy and associated with higher quality adjusted life years [14].

It is evident that the reliability of clinical history of an abrupt onset of focal or global neurological deficits such as hemiplegia, seen in more than half of the SM in this current study has its limitations. Some reports indicate that certain clinical features are highly suggestive of a SM and should be factored in when evaluating a suspected stroke patient [3, 15]. Such factors include the paucity or absence of modifiable risk factors for stroke in clinical history [3]. In our study, the presence of premorbid hypertension and increased waist-hip ratio (makers of cardiovascular disease) were statistically less prevalent among SM compared to true strokes. Further, mimics were more likely to have a normal systolic and diastolic BP on admission compared to true strokes. Finally, cortical signs such as extinction, hemianopia and supra-nuclear facial paralysis were less-suggestive of SM. Our findings are similar to what was observed by Okano et al. and Yahia et al., were a systolic blood pressure < 140 mmHg on admission was independently associated with having a SM and cortical signs were described as pathognomic for true strokes respectively [15, 16].

In this study, the use of sFABS score ≥ 3 in predicting SM among patients with suspected strokes, showed a low sensitivity (38%) and moderate specificity (80%). This contrasts with a sensitivity and specificity of 86 and 92% respectively when used in a Chinese population [10]. The low sensitivity of the scale observed in the current study could be explained by the fact that the sFABS scale was developed among those undergoing thrombolysis and therefore the current study population differs. Likewise, the demographics of the patients could be another factor; in the current study SM were elderly (mean age of 65 ± 15 years) and were therefore more likely to have lower scores. A sFABS score of ≤2 was observed in 62.4%, accounting for this low sensitivity. Our results are contrary to previous studies, were SM occur more commonly at younger ages and are less prevalent in the older population [16, 17]. Even after adjusting the cut off score to ≥2 in predicting SM, the sensitivity observed went as high as 96% with a low specificity of 46% making it unsuitable for use in clinical practice in our setting. Similarly, the biracial differences could also contribute to these discrepancies. We recommend further studies that can help develop clinical scales used for predicting SM feasible in SSA. This may help with better resource utilization, by identifying those most likely to have SM and thus benefit from imaging and other tests.

Our findings are in line with other authors, who have described brain tumors (observed in 25%) and epileptic seizures (12.5%), as the most common causes of SM [7, 15]. However, the high proportion of meningoencephalitis (16.7%) observed could be explained by the aging process which is associated with a greater propensity to contracting infections either due to physiological immunosenescence or having an underlying chronic disease.

Mortality was high at 30 days in our study, occurring in more than 50% of the patients with mimics, compared to mortality of 8.8% reported by Okano et al. [15]. The high mortality in our study could be explained by the fact that stroke mimics presented with severe disease on admission, with a mean NIHSS score of 21.7 ± 7.9 compared to a mean NIHSS score of 7.3 ± 8.9 observed in the former study [15].

Our study had the following limitations; it was a single center with a small sample size and a short follow up period therefore results cannot be generalized.

## Conclusions

In the present study the proportion of SM among patients clinically diagnosed with stroke was 6.6% and brain tumors was a common etiology. SM were less likely to have cardiovascular risk factors and cortical signs during evaluation. We thus recommend further studies that can help develop clinical scales used for predicting SM in an African population.

## Data Availability

The dataset used for analysis in this study is available from the corresponding author on reasonable request.

## Abbreviations

BP: Blood pressure
CT: Computed tomography
ECG: Electrocardiogram
FABS: Stroke mimic scoring system
IQR: Interquartile Range
MAMC: Muhimbili University of Health and Allied Science Academic Medical Center
MRI: Magnetic Resonance Imaging
mRS: Modified Rankin Scale
NIHSS: National Institute of Health Stroke Scale
SD: Standard deviation
SM: Stroke mimics
SSA: Sub Saharan Africa
STROBE: Strengthening the reporting or observational studies in epidemiology
t-PA: Tissue plasminogen activator
WHO: World Health Organization
